# Predictors for reproductive isolation in a ring species complex following genetic and ecological divergence

**DOI:** 10.1186/1471-2148-11-194

**Published:** 2011-07-06

**Authors:** Ricardo J Pereira, William B Monahan, David B Wake

**Affiliations:** 1 Museum of Vertebrate Zoology and Department of Integrative Biology, 3101 Valley Life Sciences Building, University of California, Berkeley, CA 94720-3160, USA; 2 CIBIO, Centro de Investigação em Biodiversidade e Recursos Genéticos, Universidade do Porto, Campus Agrário de Vairão, 4485-661 Vairão, Portugal; 3 Departamento de Biologia, Faculdade de Ciências da Universidade do Porto, 4169-007 Porto, Portugal; 4 National Park Service, Inventory and Monitoring Division, 1201 Oakridge Drive, Fort Collins, CO 80525-5589 USA

## Abstract

**Background:**

Reproductive isolation (RI) is widely accepted as an important "check point" in the diversification process, since it defines irreversible evolutionary trajectories. Much less consensus exists about the processes that might drive RI. Here, we employ a formal quantitative analysis of genetic interactions at several stages of divergence within the ring species complex *Ensatina eschscholtzii *in order to assess the relative contribution of genetic and ecological divergence for the development of RI.

**Results:**

By augmenting previous genetic datasets and adding new ecological data, we quantify levels of genetic and ecological divergence between populations and test how they correlate with a restriction of genetic admixture upon secondary contact. Our results indicate that the isolated effect of ecological divergence between parental populations does not result in reproductively isolated taxa, even when genetic transitions between parental taxa are narrow. Instead, processes associated with overall genetic divergence are the best predictors of reproductive isolation, and when parental taxa diverge in nuclear markers we observe a complete cessation of hybridization, even to sympatric occurrence of distinct evolutionary lineages. Although every parental population has diverged in mitochondrial DNA, its degree of divergence does not predict the extent of RI.

**Conclusions:**

These results show that in *Ensatina*, the evolutionary outcomes of ecological divergence differ from those of genetic divergence. While evident properties of taxa may emerge via ecological divergence, such as adaptation to local environment, RI is likely to be a byproduct of processes that contribute to overall genetic divergence, such as time in geographic isolation, rather than being a direct outcome of local adaptation.

## Background

In a Darwinian sense, species formation is an outcome of a continuum of gradual evolution, from ecological races and biotypes, to hybridizing taxa and, ultimately, to "good" biological species that no longer cross [1]. The long disagreement over species concepts is the best demonstration of such a continuum between populations, at a proximal scale, to the ultimate extreme of reproductively isolated species [2]. The different biological properties of species upon which several of the alternative concepts are based, such as distinct adaptive zones, fixed character traits or reproductive isolation (RI), arise at different times during the process of species formation and do not necessarily occur in a predictable order [3]. Moreover, empirical examples increasingly show that evolution of these properties does not occur in one predetermined direction. Taxa may remain genetically isolated without approaching full RI, as, for example, by forming hybrid zones maintained by a balance between dispersal and selection.

Alternatively, if selection is strong, reinforcement of reproductive barriers may lead to full RI (e.g. green-eyed tree-frog [4]) or, if the strength of selection decreases due to environmental change, gene flow might fully reverse the differentiation process by homogenizing the parental taxa into a single gene pool (e.g. three-spined stickleback [5]; cichlids fish [6]). In spite of the ongoing discussion of the relative importance of RI in the continuum that culminates in the formation of new species (see [1]), there is a consensus that the effect of RI is to cause divergence along that continuum. Therefore, once RI is complete (establishment of post-zygotic mechanisms barring successful genetic interactions [7]), the evolution of closely related taxa is no longer reversible and they are bound to follow independent evolutionary trajectories, even in the face of environmental change.

Reproductive isolating mechanisms are generally understood to accumulate gradually as a function of molecular genetic distance, a surrogate for time since divergence [8]. Substitutions that are neutral or beneficial on one genetic background may be deleterious on another, and postzygotic isolation may often reflect such negative epistatic interactions. Experimental studies strongly favor this view, showing that mutations in coevolving gene complexes can rapidly cause hybrid incompatibilities in closely related species [9].

Alternatively, non-neutral processes such as ecological divergence may incidentally cause RI [10-12], either due to pre- or post-zygotic mechanisms. If fitness is habitat-dependent, rather than a consequence of the overall genomic composition, it would be lower for immigrants from alternative habitats or for individuals that are intermediate for ecologically relevant traits. Two central predictions of ecological species formation [13] are 1) ecologically divergent pairs of populations will exhibit greater levels of RI than ecologically similar pairs of populations of similar age; and 2) traits under divergent selection, or those genetically correlated with them, should incidentally affect RI (e.g. mate preference, hybrid fitness). The recent accumulation of ecological and genetic data in natural populations enables this hypothesis to be formally tested using comparative approaches that separate the effects of genetic and ecological divergence. Meta-analyses across plant and animal taxa that statistically remove the effect of time since divergence show that, as the degree of ecological divergence increases, so too does their degree of RI [14]. However, this study relies on broad taxonomic comparisons across plant and animal species, which are neither historically nor biologically related. This hypothesis has been further evaluated using comparisons of closely related units with known genealogy, extending that approach to a phylogenetic scale that is more appropriate for the scale at which RI develops (i.e. intra specific level). However, this has only been tested in organisms that experienced parallel evolution, in which ecological divergence between sympatric populations repeatedly results in assortative mating, resulting in taxa with little or no genetic differentiation (e.g. sticklebacks [15], and *Gambusia *fishes [16]). Yet, these results might not be generalizable to taxa that do not evolve *in situ*, such as those in which geography is an important component of the process of species formation leading to spatial fragmentation, genetic and ecological divergence, and secondary contact.

Ring species were initially hypothesized as a single species that expands along two pathways around a geographic barrier, with terminal forms gradually diverging and eventually behaving as two species when they meet on the other side (Stejneger in [17]). The persistence of two reproductively isolated forms, connected by a chain of intermediate populations, demonstrate the linkage between micro-evolutionary processes and formation of reproductively isolated taxa, i.e. "good" biological species. Only a few species complexes are known to meet these criteria, including *Ensatina eschscholtzii *[18], *Phylloscopus trochiloides *[19], and *Platycercus elegans *[20]. In spite of the varying degrees of regional extinction or present connectivity between intermediate populations, all these examples are natural demonstrations of the continuum between population- and species-level divergences. Therefore, ring species are excellent candidates for studies of genetic interactions at different stages of divergence, in a natural setting, and for investigating how species properties such as RI might arise.

**Figure 1:**
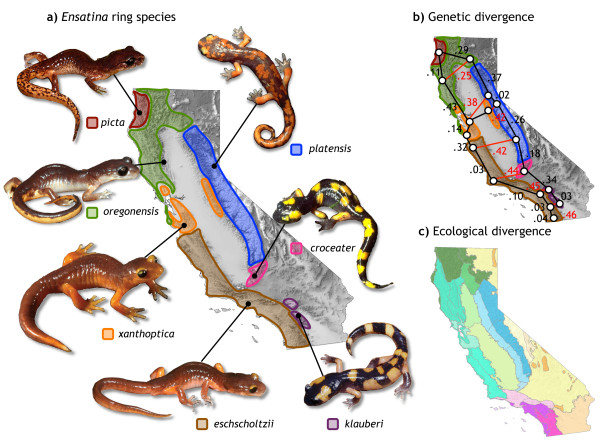
**Divergence processes in the ring species *Ensatina eschscholtzii***. a) Ecomorphotypes in *Ensatina *defined by color pattern. b) Nei's (1972) genetic distances based in 26 allozyme loci (adapted from [34]), showing increased differentiation towards the terminus of the ring (red) and lower genetic divergence between populations around the ring (black). c) Floristic provinces of California (adapted from [30]); different colors refer to distinct plant associations.

The plethodontid salamander *Ensatina eschscholtzii *is a well characterized ring species complex, with an almost complete ring distribution. Gradual genetic and morphologic transitions occur between intermediately derived forms around the ring, whereas abrupt changes occur between the most divergent forms at the terminus (Figure 1a), with rare hybridization restricted to few generations of hybrids or even full RI [21,22]. The complex originated in northern California, probably during the late Miocene [23-25], and expanded southwards around the Central Valley, which constitutes a long standing geographic barrier, first as a lake and more recently as ecologically unsuitable habitat. During the climatic change of the Holocene [26], a presumed temporary corridor across the Central Valley enabled the coastal populations to colonize the foothills of the Sierra Nevada, providing a second closure of the ring approximately midway along the ring distribution. Molecular studies brought strong support for the colonization around the Central Valley (Figure 1b), but also indicate that historical periods of geographic isolation, more or less prolonged, occurred between populations around the ring, due to the dynamic geographic history of California [21,24,27]. These studies suggest that time since divergence might have strongly contributed to the continuum of genetic interactions observed in the ring species, but the contribution of ecological factors promoting local adaptation in *Ensatina *remains to be evaluated.

As the species expanded its range, it colonized habitats in the coastal and inland ranges of California that are substantially different in ecology. Distinctive and different color patterns resulted from the evolution of alternative predator avoidance strategies [18,28,29] that are more extreme at the southern closure of the ring. The distinct color patterns are presently recognized as seven subspecies (Figure 1a) that do not correspond to the deepest genetic breaks [22], suggesting that the evolution of coloration cannot be explained by neutral processes (mutation and drift). Moreover, this high phenotypic variability in color pattern is strongly regionalized in areas with ecologically similar habitat (Figure 1c; [30]). Cryptic and mimetic color patterns are hypothesized to be advantageous with respect to predation in their own habitats, resulting in strong selection against inter-population migration or hybridization between alternative color morphs [29]. Field experiments support fitness differences for alternative color patterns [31], and strong selection occurs at a hybrid zone between taxa with mimetic and cryptic coloration in the area of the mid-closure of the ring [32]. These facts suggest that the ecologically diverse landscape of California plays an important role in the diversification of this complex and in the limitation of genetic interactions among lineages [29]. Thus, in addition to the genetic differentiation around the Central Valley, adaptation to local habitat conditions might also be correlated with the continuum of RI observed in this ring species.

**Figure 2:**
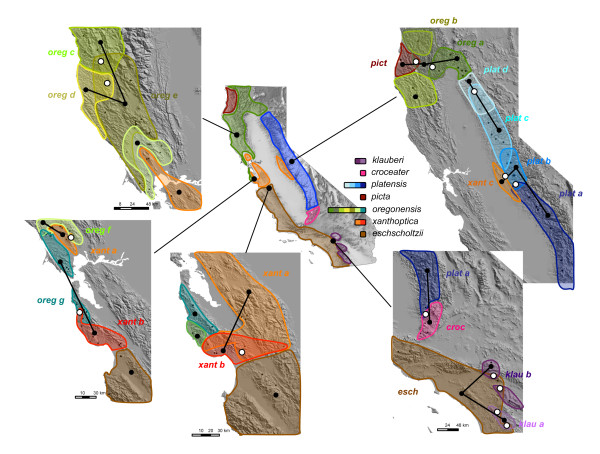
**Sampling of secondary contacts in the ring species *Ensatina eschscholtzii***. The distribution of *Ensatina *in California is colored according to the assignment of individuals collected throughout its entire range and genotyped for 22 to 27 allozyme loci (see [22] for details). The seven main colors (inset) correspond to the distinct color patterns, taxonomically recognized as subspecies. Different shades of those main colors refer to the genetically distinct units identified within each subspecies (outset), and were drawn considering the geographic coordinates of sampling. Localities with genetically admixed individuals or sympatry of pure forms are represented by overlapping ranges of the pure parental populations. Black dots mark the 20 parental populations, black lines mark the 13 pairwise comparisons considered in this study, and white circles mark the localities used to estimate degrees of genetic admixture at each contact zone.

Episodes of geomorphological and climate change led to varying degrees of geographic isolation and ecological divergence around the ring, in particular to the ring-like geographic range with mid-way and terminal closures, offering a natural replication of secondary contacts along the continuum of species formation. Previous work showed an association between genetic differentiation in allozymes and RI, but did not consider whether other co-occurring indices of genetic divergence (such as mtDNA) and major ecological factors known to be important for *Ensatina *might explain the observed levels of RI. We take advantage of the multiple contact zones in the *Ensatina *ring species (Figure 2) to employ a quantitative framework to infer the processes restricting genetic interaction upon secondary contact, which may result in full RI. Using previously published datasets on allozymes from all extant populations around the California's Central Valley [22], we distinguished allopatric populations that are not affected by recent introgression from regions of secondary contact where localized genetic introgression occurs. By augmenting genetic datasets and collecting new ecological data on the allopatric ranges, we now estimate levels of genetic and ecological divergence in all populations, prior to secondary contact. By measuring the frequency of genetically admixed individuals at the center of each secondary contact we estimate the degree of RI between pairs of parental population contacting around and across the ring species distribution, and how it is predicted by the several axes of divergence between parental populations. Our results indicate that the isolated effect of ecological divergence between parental populations does not result in reproductively isolated taxa. Instead, processes related with overall genetic divergence are the best predictors of reproductive isolation.

## Results

### Sampling and study units

Although most of the 20 genetically distinct populations interact in zones of secondary contact around the Central Valley [22], there are a few gaps around the ring that either represent real distributional gaps (e.g. Mohave Desert) or possible sampling deficits (e.g. Lassen Peak, Pajaro river). Our sampling detected 13 contacts around the ring, plus one at the mid-ring and two others at the terminus, for a total of 16 contacts. However, because three of the contacts around the ring involved parental populations with low genetic divergence (*D*_N _< 0.1), we consider a total of 13 contacts in our analysis of RI (Table 1). These contacts involved 19 different pure parental populations, of which 15 were used in a single pairwise comparison, and only four were used in two comparisons (Figure 2).

**Table 1:** Predictor and response variables for development of reproductive isolations in the ring species *Ensatina eschscholtzii*.

Population comparison	*N*	Response Variable	Predictor Variables			
		
		Degree of Genetic Admixture (%)	Genetic Divergence		Ecological Divergence	
			
			Time since Divergence		Climatic dissimilarity (%)	Vegetation dissimilarity (%)
			mtDNA distance (corrected PiXY)	nDNA distance (*D*_N_)		
**contacts around the ring:**						
*plat a vs croc*	9	88.9	2.9	0.11	51.9	82.9
*plat a vs plat b*	6	100.0	8.6	0.25	82.5	16.4
*plat c vs plat d*	11	81.8	5.5	0.17	100.0	33.3
*oreg a vs oreg b*	8	87.5	6.8	0.17	71.3	36.1
*oreg b vs pict*	23	56.5	3.1	0.39	93.1	47.6
*oreg c vs oreg e*	10	100.0	1.6	0.13	100.0	39.4
*oreg d vs oreg e*	8	100.0	11.0	0.16	85.3	32.0
*oreg f vs xant a*	6	100.0	13.8	0.31	80.2	29.3
*oreg g vs xant b*	8	50.0	11.0	0.21	58.0	29.0
*xant a vs xant b*	9	75.0	5.6	0.12	100.0	13.8
**mid-ring contact:**						
*xant c vs plat b*	323	35.9	12.9	0.4	86.3	4.6
**contacts at the terminus of the ring:**						
*esch vs klau b*	18	11.1	10.1	0.56	94.7	60.3
*esch vs klau a*	88	5.7	10.5	0.6	100.0	24.0

### Contributors to Genetic Divergence

As expected given the known phylogeography of the *Ensatina *complex based on mtDNA [24,33], genetic distances in nDNA are in agreement with the ring species scenario [21,34]. Populations that diverged along the same side of the Central Valley are more similar in nDNA, compared to those that diverged on opposite sides of the ring. The new individuals sequenced for cyt *b *recover some new sequences nested within the clades already described for the whole species complex [24]. The parental populations as defined by allozymes do not share any haplotype in mtDNA. However, the degree of genetic divergence in mtDNA across secondary contacts does not match that in nDNA, showing that these two genetic distances are not correlated. While the higher nuclear genetic distances only occur between taxa that contact across the ring, higher genetic distances in mtDNA are found between coastal populations within the ring (Table 1).

### Contributors to Ecological Divergence

**Figure 3:**
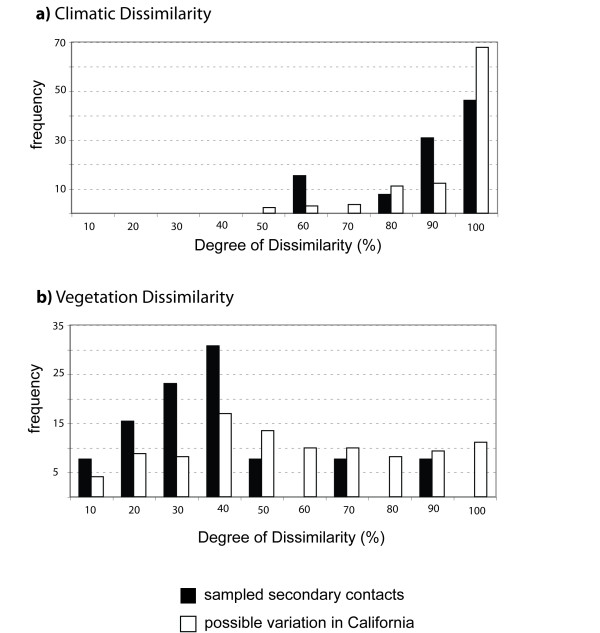
**Indices of climatic (a) and vegetation (b) dissimilarity between populations of *Ensatina *within California**. White bars correspond to all possible pairwise comparisons between the 20 populations and reflect the underlying ecological variation intrinsic to the geographic area occupied by *Ensatina *in California. Black bars correspond to the dissimilarity indices from pairs of populations that contact around or across the ring distribution.

In California, the climatic space occupied by *Ensatina *as defined by PC1 and PC2 captures 73.7% of the variation in the entire distributional data (Additional file 1). Pure parental populations that contact around or across the ring distribution of *Ensatina *have little or no overlap in climatic space (Figure 3a, black bars). However, the degree of dissimilarity between the focal comparisons of parental populations matches the underlying climatic variation intrinsic of California (Figure 3a, white bars). Despite the heterogeneity of climatic space in California, the highest degrees of climatic dissimilarity between *Ensatina *populations are found in northwestern California, along the Sierra Nevada, and near the terminus of the ring distribution (Table 1).

In contrast with climate, the focal parental pairs of *Ensatina *are generally associated with similar types of vegetation (Figure 3b). This pattern does not reflect the background variation of vegetation in California, which has areas with very similar and very dissimilar vegetation. A few of our focal contacts occur between populations with high dissimilarity in vegetation (Table 1), such as in the northwestern coast of California and in southern Sierra Nevada.

### Degree of Genetic Admixture in secondary contacts

The frequency of admixed individuals fits our expectation for a ring species (see also [21,22]). In contacts around the ring we generally found a high frequency of hybrids (above 75%), with a couple of exceptions between groups that are genetically and morphologically divergent (Table 1). Reproductive isolation is nearly complete at the southernmost contact across the ring, where 5.7% of 88 individuals sampled in sympatry were hybrids. In the mid-ring contact, the frequency of hybrids (35.9%) was intermediate to values found in contacts around the ring and at the terminus. The hybrid individuals sampled in the 13 secondary contacts consist almost entirely of backcrosses of second or older generations (not assigned to F1 or first generational backcross with *pp*> 0.9). The exceptions are at the two terminal contacts of the ring, where the few hybrids detected (11.1 and 5.7%) are assigned to F1 hybrids or first generational backcrosses, suggesting that in addition to the nearly complete level of RI between parental populations, hybrid individuals rarely reproduce and therefore are not contributing for gene flow between parental populations.

### Model for Reproductive Isolation

**Figure 4:**
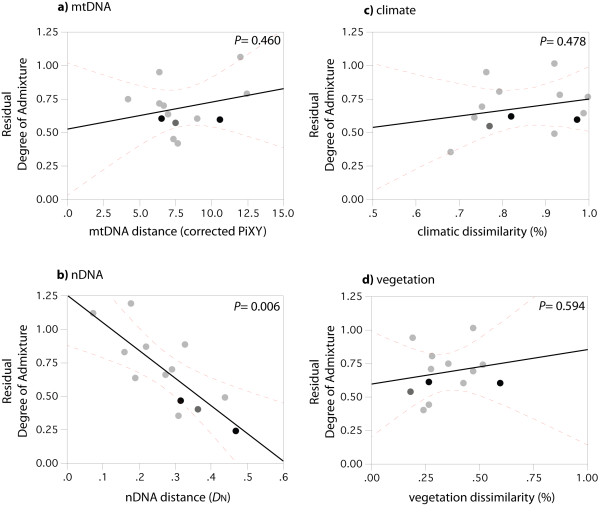
**Isolated effect of each of the four predictors for the development of reproductive isolation in the *Ensatina *ring species: a) mitochondrial DNA, b) nuclear DNA, c) climate, and d) vegetation**. Data points are pairwise comparisons between populations that contact around the ring (ten light grey points), at the mid-ring (one dark grey point) and at the terminus (two black points). Solid black lines represent the best-fit line for each predictor variable, dashed red lines designate contours of the predicted formula, and respective probabilities are denoted in the corner of each plot.

When the four kinds of divergence between pairs of parental populations (in mitochondrial and nuclear DNA, climate and vegetation) are taken together in a multiple regression as independent variables, we obtain a model that predicts the degree of genetic admixture observed within *Ensatina *ring species (R^2^= 0.733, *P*= 0.02). After statistically correcting for the correlation between predictors (Figure 4), genetic distance, as determined in nDNA, is the only predictor that shows a prominent negative relationship with the degree of genetic admixture (slope = -2.063 +/-0.549, mean +/- se, t_13, 0.05 _= -3.76, *P *= 0.006), as expected for divergence processes associated with the development of RI. The isolated effect of ecological dissimilarity (both in climate and vegetation) and mitochondrial divergence is not associated with a decrease of genetic admixture in secondary contacts.

## Discussion

We predicted that divergence in properties of parental populations that cause RI, or that are associated with them, should be correlated with a decrease in the degree of genetic admixture, i.e. percentage of hybrids in contact zones. Traits of parental populations related to genetic divergence, particularly nuclear genetic distance, are the best predictors of RI. In contrast, divergence in the ecological traits measured here (climate and vegetation) is unlikely to result in reproductively isolated units. Previous analysis using data from species pairs across plants and animals [14], after statistically removing the effect of time (genetic distance), found ecological divergence to be associated with RI. However, we did not detect such an association within *Ensatina*, which suggests that taxa subjected to ecological divergence may fail to achieve complete RI. Instead, the complete cessation of genetic interactions between closely related taxa is strongly associated with the overall genetic divergence in nuclear markers, which likely reflect selectively neutral processes.

### Discordance between genetic divergence in mt and nDNA

The degree of mitochondrial divergence is a widespread criterion in phylogeography that is often used to make predictions about the evolutionary independence of lineages and ultimately their taxonomic status. Even though simulation and empirical studies strongly discourage applying single-gene criteria for species discovery (e.g. [35]) this approach has motivated ongoing data collection across the entire tree of life (i.e. "DNA-barcoding"). In our comparative study within *Ensatina*, while we find a good qualitative agreement between mitochondrial and nuclear markers (i.e. major mtDNA lineages encompass distinct nDNA genetic clusters), we also find a quantitative disagreement in the depth of genetic divergence across pairs of populations (Figure 4a and 4b). Thus, the degree of divergence in mtDNA does not predict the degree of RI between parental populations, whereas average divergence across nuclear markers does.

Whereas mitochondrial genetic distances effectively reflect the evolutionary history of a single non-recombining locus, the multi-locus perspective from nuclear genetic distances was calculated using 22 to 27 physically unlinked allozyme loci [22]. In addition, the mitochondrial molecule has an effective population size four times lower than the nuclear markers, and therefore is more subjected to the effect of genetic drift, leading to a rapid fixation of the most common alleles in populations that are spatially fragmented or that experience low migration rates. An alternative explanation is that mtDNA is not evolving neutrally and therefore does not reflect time since divergence in geographic isolation. Studies in allopatric ranges of *Ensatina *populations do not detect molecular signatures of selection in mtDNA [24]. However, studies of secondary contacts show that steep parapatric transitions between mitochondrial lineages are accompanied by leaky borders for allozymes [22,32], suggesting that selection might prevent introgression of the mitochondrial genome.

Although obtaining mtDNA data is fast, inexpensive and is a useful source of genetic information on population structure, conclusions about the degree of RI drawn from mitochondrial genetic distances can be misleading. In contrast, information on the degree of divergence determined using several autosomal loci is more informative in predicting the degree of genetic interactions in secondary contacts.

### Ecological Divergence as a factor in Reproductive Isolation

Adaptation to distinct environments via natural selection is doubtless a major process that can drive species diversification [36], and it has deservedly received attention from both theoretical and empirical biologists (see [37,38]). In association with development of the modern synthesis and the biological species concept, RI became regarded as the main property of species that would assure genetic integrity, and therefore evolutionary independence [39,40]. RI evolves as the pleiotropic effect of loci under divergent natural selection, or the direct effect of loci in linkage disequilibrium [41]. Therefore, a central prediction of ecological species formation is that ecologically divergent pairs of populations will exhibit greater levels of reproductive isolation than ecologically similar populations of the same age [13].

Integrating ecological and genetic data into the framework used here has provided a powerful way to evaluate the relative contribution of genetic and ecological divergence for the development of isolating mechanisms between closely related taxa (see also [13,14,42]). By analyzing ecological and genetic data from inter-specific comparisons, across plants and animals, Funk et al. [14] found an association between ecological divergence and RI, after correcting for genetic divergence. However, when we extend that same approach to intra-specific comparisons within the *Ensatina *ring species, no such association is detected (Figure 4). Our results should be taken as an indication that generalizations are premature, and that for taxa in which geography conditions genetic and ecologic divergence, the development of RI might differ from the known examples of parallel speciation [15,16]. Polytypic species and species complexes provide a multitude of comparisons of units of known genealogy, and enable testing the hypothesis at various temporal scales during the progression of species formation.

The selected ecological predictors, climate and vegetation, are proxies for two distinct processes that are putatively important for the diversification of *Ensatina*, respectively adaptation to physiological challenges and specialization to particular habitats. In other systems, divergence in color pattern is known to lead to RI due to physical genetic linkage with loci controlling for assortative mating [43]. Also, divergence in diet or micro-habitat choice is known to affect RI by pleiotropic effects [44]. At the spatiotemporal scale considered here, divergence in climate and vegetation is not associated with a decrease of genetic interactions upon secondary contact (Figure 4). Accordingly, their isolated effect does not result in RI barriers. However, both color pattern and habitat choice are likely to constitute complex traits in habitat-dependent organisms such as *Ensatina*. Thus, it is possible that both climate and vegetation might affect RI at a finer spatial scale, more proximate to the scale at which the organism uses the habitat (e.g. a few meters), or at an older time scale, during which vicariance within the ring species complex likely occurred (e.g. during glacial cycles). The possibility remains that other ecological parameters not evaluated here, such as local predators or microhabitat characteristics, might be associated with the evolution of RI. Testing those new hypotheses will require extending this approach to other ecological factors and spatial scales appropriate to the population biology of this salamander, such as neighborhood size and dispersal rate.

### Ecological Divergence and genomic heterogeneity

Ecological diversification without complete RI is commonly observed in nature (e.g. *Heliconius *butterflies [45], cichlid fishes [6], Darwin finches [46]) and is often considered an indication of "incomplete speciation" [42]. Our results in *Ensatina *question the almost generalized expectation that RI is also a property of species formed by ecological divergence, and support the observation that "ecological species" might not develop complete reproductive barriers [47]. Despite the lack of RI, divergent natural selection on traits between environments is likely to result in other properties that are generally assigned to species, such as morphological diagnosability, an ecological niche, a stable geographic range, and eventually genetic distinctiveness in neutral loci. However, RI might be a product only of geographic isolation, rather than a direct consequence of divergent selection for ecologically relevant traits. Spatial contact between ecologically divergent taxa, rather than resulting in RI, might more often lead to the formation of hybrid zones (i.e. stable boundaries between hybridizing taxa), which are generally located in ecotones where individuals with parental traits will have intermediate fitness and may co-occur [48]. As long as divergent selection between parentals remains stable, parental taxa, or the properties we recognize in them (e.g. phenotype, genetic diagnosability at certain loci), are preserved without approaching complete RI.

The lack of RI in "ecological species" might not represent a lack of integrity or stability through time. Hybrid zones are likely to constitute important selective filters to gene flow, allowing locally adapted alleles to remain in the environments where they are most fit, while neutral alleles might introgress. Reports of genomic divergence across sister species that diverged ecologically show a consistent pattern of highly heterogeneous genomes (e.g. [49-52]). This is hypothesized to happen because the few genes under selection or physically linked to loci experiencing strong disruptive selection can diverge, whereas gene flow will homogenize the remainder of the genome, resulting in isolated "genomic islands of speciation" (e.g. [49]). At an extreme, genomes of sister ecological species might be completely homogeneous, with the exception of those areas that initiated ecological divergence (e.g. *Heliconius *butterflies [53,54]).

The genic view of species postulates that "species are groups that are differentially adapted and, upon contact, are not able to share genes controlling these adaptive characters, by direct exchanges or through intermediate hybrid populations. These groups may or may not be differentiated elsewhere in the genome" [55]. Our results are in agreement, and suggest that ecological divergence *per se *does not result in reproductively isolated taxa. The patterns of clinal genetic variation or weak genotypic clustering commonly found between ecologically divergent taxa (ecological species) in nature probably result from processes similar to those in *Ensatina*. The fact that we consider species formation to be "incomplete" results only from our assumption that species will only endure in time in the presence of RI, rather than being genetically isolated in most of their genomes, or particular genomic regions controlling for ecologically relevant traits. Hybrid zones between ecological taxa (species, races or populations) where transition in ecological traits remains abrupt despite the lack of complete RI or absolute restrictions to gene flow, constitute the evidence that RI is not necessary, or even advantageous, for ecological differentiation. While it is irrefutable that natural selection is an important force in species diversification, that process may well be uncorrelated with the development of RI.

## Conclusions

Since the time of the modern synthesis, we have made major progress in understanding how RI may arise in various geographic contexts (sympatric, parapatric, allopatric). Whereas RI is the ultimate test as to whether two populations should be considered species, this process only reflects a single axis of the whole process of species formation. This limitation has been both technological and conceptual. However, recent technological advances now allow us to operate not only at the level of the organism, but also on the genes responsible for the traits we attribute to species, and how they interact with the local environment. Extending this same approach to other response variables that are now more tractable, such as restriction of gene flow in selected versus neutral loci, and comparison across several hybrid zones with different levels of divergence, will soon enable us to break from assumptions of what a "good" species should be, and directly illuminate the multidimensional nature of species formation.

## Methods

To determine the best predictors for the development of reproductive isolation we followed a three-step approach. First, we distinguish the range of pure parental populations (i.e. not affected by ongoing gene flow) from areas of secondary contact (i.e. where genetic introgression occurs). Second, we focus on the ranges of pure parental populations to infer the amount of genetic and ecological divergence (predictor variables) prior to secondary contact and without the confounding effect of genetic and ecological intermediates. Third, we focus on individuals collected at the center of secondary contacts to determine the frequency of genetic admixture (response variable) and statistically infer relationships between predictors and the response variable.

### Sampling and study units

Because we want to predict how reproductive isolation (RI) develops along the whole continuum of species formation, our operational units need to be at any diagnosable stage of divergence along the gradient from genetically distinct populations, ecomorphological groups (subspecies), and reproductively isolated taxa. In *Ensatina*, ecologically and morphologically distinct groups are subdivided into several genetically distinct units, with varying degree of genetic divergence [22]. Therefore, we chose to discard any a priori taxonomic status and operate with every kind of genetically distinct population. A population is here defined as a genetically homogeneous group of individuals that is in mutation-drift equilibrium, irrespective of their spatial range or the extent of genetic differentiation between them.

The range of a ring species complex comprises areas of genetic homogeneity, where genetically pure parental individuals occur, linked by areas of secondary contact, where genetically admixed individuals occur as a consequence of recent genetic interactions. In the *Ensatina *complex, as in other organisms with low dispersal, genetic variability has a fractal spatial structure [24,27]. Morphologically homogeneous groups (subspecies) that occupy a large range can be subdivided in smaller genetically homogeneous groups, which, using appropriate genetic markers, might be further dissected into families or even individuals. Patterns of genetic variation of 22-27 allozyme loci, in 1130 individuals collected throughout the entire ring distribution, enable recognition of at least 20 genetically distinct "parental populations" around the Central Valley [22]: 2 within *klauberi*, *croceater*, 4 within *platensis*, 8 within *oregonensis*, *picta*, 2 coastal plus a third inland population of *xanthoptica*, and *eschscholtzii*. The individual multilocus genotypes were assigned to groups maximizing Hardy-Weinberg (HW) and linkage equilibria at a uniform level of diagnosability (see [22] for details), using the Bayesian clustering algorithm implemented in the software STRUCTURE [56].

In this study, we use the population structure analysis from Pereira and Wake [22] to distinguish the ranges of the 20 genetically pure parental populations, which are not affected by ongoing gene flow, from the areas of secondary contact around and across the ring, where genetic admixture occurs. Using assignment tests, genetically pure individuals are assigned to a single cluster with high posterior probability (*pp *close to 1, while admixed individuals have intermediate *pp *of belonging to two clusters (e.g. F1 hybrid would have 0.5 *pp*). For purposes of this study, localities with a mean assignment to a single cluster higher than 0.8 *pp *were considered "parental localities", while those with assignments lower than 0.8 *pp *were considered "admixed localities". Polygons encompassing parental localities represent the range of genetically pure "parental populations" (*N *= 20), whereas intervening polygons represent areas of "secondary contact" (*N *= 16; Figure 2). The spatial boundaries of those polygons were robust to variation around the 0.8 *pp *threshold.

In addition to these previously published genetic data [22], we include data from two other studies that focus on the midrange closure of the ring, between inland *xanthoptica *and *platensis *(Additional file 2). To determine the amount of genetic divergence, we used new data from 121 individuals sampled within the range of genetically pure populations along the Sierra Nevada (23 geographic localities), genotyped for 24 allozyme loci. To estimate the degree of genetic admixture we used published data from three independent transects on that hybrid zone [32], which included 323 individuals genotyped for 8 allozyme loci diagnostic for those parental populations.

Subsequent to this analysis, genetic data from individuals collected within the range of "parental populations" and individuals collected within areas of "secondary contacts" were treated in separate datasets to measure genetic divergence and degree of admixture independently.

### Contributors to Genetic Divergence

In a ring species, the accumulation of genetic divergence depends both on the bidirectional colonization around the main geographic barrier, and on restrictions to gene flow caused by periods of geographic isolation. At the level of this analysis, we cannot always differentiate between the effect of time and space, but both factors are known to affect the diversification process in *Ensatina*. Phylogeographic studies using mitochondrial DNA (mtDNA) support the ring species hypothesis, in particular the pattern of clade origin in northern coastal California, and southwards colonization around the Central Valley [24,33]. Moreover, since the beginning of its diversification in the late Miocene, *Ensatina *has been subjected to the dynamic geologic history of California. The formation of island isolates together with the movement of tectonic plates resulted in periods of geographic isolation having varying lengths between populations around the ring [21]. As a consequence of time in geographic isolation, high genetic differentiation exists in allozymes and mtDNA between morphologically similar populations that contact across previous geological barriers [21,22,24]. We recognize that genetic divergence among populations is a function of divergence time and historical introgression (i.e. prior to the current secondary contact). Nonetheless, overall genetic divergence should be a valid predictor of current RI, as estimated from the frequency of hybrids in the present contact zones [8]. Here, we use two measures of genetic distance commonly used in the literature: divergence at one mitochondrial gene, and divergence averaged across several nuclear loci.

#### a) Genetic distance in mitochondrial DNA

In order to determine divergence in mtDNA, we analyzed genetic variation in an 802 bp fragment of cytochrome *b *(cyt *b*) from 344 salamanders sampled in 212 unique sites within the range of the 20 pure parental populations. We used previously published data from 263 individuals [24] (GenBank accession numbers FJ151653 - FJ152002; see Additional file 3) and sequenced another 81 individuals following the same protocol to represent all extant populations of *Ensatina *and intra-population variability. Sequences were visually aligned in SEQUENCHER (Gene Codes, Ann Arbor, MI, USA) and deposited in GenBank (accession numbers JN022615 - JN022695). We calculated corrected average pairwise differences between pure parental populations (PiXY [57]) using the Tamura-Nei model of sequence divergence implemented in ARLEQUIN v.3.11 [58].

#### b) Genetic distance in nuclear DNA

To estimate divergence across nuclear genes (nDNA), we used the 22 to 27 allozymic loci described above, which are selectively neutral based on Hardy-Weinberg equilibrium [22] and are not likely to directly affect reproductive isolation. Because genetic divergence decreases due to gene flow after secondary contact, we restricted this analysis to our genetically pure parental populations, by combining all the "pure" individuals assigned to the same population (assignment *pp≥*0.95) and calculating Nei's *D *[59] with GENETIX [60].

### Contributors to Ecological Divergence

During the southwards expansion of the complex, *Ensatina *colonized disparate habitats throughout California, from wet and cold climates characteristic of northern California, to arid habitats in the southern part of the range. These steep climatic gradients are likely to constitute important physiological challenges for ectothermal and lungless organisms such as *Ensatina*. Moreover, California is marked by distinct physiographic regions that are characterized by specific plant communities [30]. Although the distribution of *Ensatina *is contiguous across most vegetation types, changes in color pattern, and therefore subspecies limits, are spatially concordant with turnover of vegetation composition. We here account for the putative effect of ecological divergence by independently evaluating the effect of climate and vegetation, since they would be attributed to distinct ecological processes important to *Ensatina *and are generally recognized as major ecological factors that could drive RI.

We evaluated ecological divergence with respect to current climate and vegetation using the distribution of *Ensatina *around the main geographic barrier that defines the ring, the Central Valley. We gathered observation points of *Ensatina *in California by compiling information from field records at the Museum of Vertebrate Zoology (UC Berkeley) and surveying herpetological collections from most North American natural history museums using HerpNET on 5 May 2009. From a total of 12,366 observations, we accounted for sampling biases by reducing the number of points to spatially unique samples - defined according to the georeferences associated with the specific localities of the specimens. Samples were assigned to pure parental populations or contact zones in between, using polygons defined by the genetic data (above). We then queried the samples for 19 continuous bioclimatic variables (1-km^2 ^spatial resolution [61]) and 55 binary vegetation variables (0.01-km^2 ^spatial resolution, California Wildlife Habitat Relationships System). Ecological divergence was calculated based on 3,404 assigned spatially unique observations of *Ensatina *around the Central Valley of California. Because we did not find associations between these traits and RI, we did not consider possible confounding effects of interaction between climate and vegetation any further.

#### a) Climatic Dissimilarity

We first described the entire climatic space occupied by *Ensatina *in California using a principal component analysis (PCA) on the 19 bioclimatic variables, which includes records in areas occupied by parental populations and contact zones. Using the first two principal components (PC axes), we then calculated the area of climatic space occupied by each parental population. Using the subset of records assigned to each of the 20 pure parental populations, we calculated their respective climatic areas by using a kernel density function [62] (bivariate normal kernel, 95% density, 500 × 500 pixel grid); this method of estimating area in the PCA was preferable to a minimum convex polygon, which would have included large areas devoid of any sampling points. Finally, we computed the percentage of area overlap in climatic space between each focal pair of parental populations, where overlap was defined as the area intersect divided by the area union. Climatic dissimilarity was calculated as 1- climatic overlap and expressed as a percentage, so that 0% corresponds to population pairs with the same climate, and 100% to the populations occupying completely dissimilar climates.

#### b) Vegetation Dissimilarity

To estimate vegetation divergence between pure parental populations, we again used a dissimilarity index, here based on the presence or absence of every vegetation variable or class. For each pairwise comparison between populations, we first calculated the percentage of the number of localities of *Ensatina *that were associated with the vegetation classes unique to either of the two focal populations. We then divided this tally by the total number of localities in the two populations, expressed as a percentage. This generated vegetation dissimilarity values ranging from 0% for populations associated with identical vegetation variables to 100% for populations associated with completely different vegetation variables.

### Degree of Genetic Admixture in secondary contacts

As a proxy for the development of reproductive isolation, we now focus on the individuals collected at secondary contacts to measure the degree of genetic admixture observed at contact zones at the several levels of divergence present around and across the ring complex. When RI between parental taxa is complete, or almost complete, due either to pre- or post-zygotic mechanisms or both, we expect that most of the individuals in areas of sympatry will genetically resemble those from parental populations. On the other hand, if isolating mechanisms are not fully developed, we expect that a few generations of random mating will quickly produce admixed individuals that are genetic mosaics of more than one parental genome (herein "hybrids"). Here, the use of the word "hybrid" referring to a genetically admixed individual does not imply any a priori knowledge about the degree of RI between the respective parental taxa, but only that those taxa are genetically diagnosable at the level of our analysis.

We assessed the frequency of genetically admixed individuals based on their multilocus genotypes for the allozyme data described above, using the Bayesian assignment test implemented in the program NEW HYBRIDS [63]. This method uses multilocus genotypes to assign each individual to genetic categories (i.e. parental A, parental B, F1 hybrid, or backcrosses), without a priori assumptions of their ancestry or location, while it estimates allele frequencies in parental populations trying to maximize HW and linkage equilibria. To calculate the degree of genetic admixture between each pair of populations we classified individuals from the sampling locality in the center of each secondary contact (see Table 1) into genetic categories, using a threshold of 0.9 *pp*, and calculated the frequency of hybrids (F1 and backcrosses).

Because the program works under a two-population model, we performed individual runs for each contact, including the samples from localities within the secondary contact as our query sample, and from localities in the range of both parental populations as reference samples for the assignment. Because the limits of some contact zones are better defined as a genetic gradient rather than an abrupt change, we did not discriminate our reference samples as genetically pure (*z *and *s *options off, see manual), basing the assignment solely on the multilocus genotypes. We pseudo-replicated the Markov chain Monte Carlo from different starting points and ran the analysis long after reaching stability to assure convergence to the same result. Although the assignment test implemented in NEW HYBRIDS is mainly driven by HW and linkage equilibria, genetic differentiation between parental populations is known to affect the power of discriminating admixed individuals. Therefore, we did not compute frequency of admixture in secondary contacts between populations that diverged less that 0.1 (*D*_N_) in allozyme loci. Because we have more than one transect across some hybrid zones (contact zones across the ring), we calculated the frequency of hybrids by pooling individuals and computed means across the multiple transects.

### Model for Reproductive Isolation

Because genetic and ecological differentiation between parental populations often co-occur, we used the multiple contacts within the ring species to statistically isolate the association between different processes that might result in RI [42]. We employed a multiple regression model, implemented in the statistical package JMP 5.0 (SAS Institute, Inc., Cary, NC, USA), by defining the degree of genetic admixture as the response variable, and the four kinds of distances or dissimilarities between parental populations as the predictors (genetic distance as measured by mtDNA and nDNA, and dissimilarity in climate and vegetation). By using leverage or partial residual plots to examine the isolated effect of each predictor variable on the degree of genetic admixture, we expect to distinguish which processes are associated with the evolution of reproductive barriers between taxa. We predict that divergence in traits of parental populations that cause RI, or in those associated with them, will be negatively correlated with the degree of genetic admixture.

## List of abbreviations

RI: reproductive isolation; HW: Hardy-Weinberg; mtDNA: mitochondrial DNA; nDNA: nuclear DNA; PC: principal component.

## Authors' contributions

RJP carried out the design of the study, compiled published genetic data, collected new genetic sequences, analyzed the data, interpreted the results and drafted the manuscript. WBM collected the ecological data, preformed spatial analyses and participated in the interpretation of the results. DBW contributed with new allozymic data and interpretation of the results. All authors participated in the preparation of the manuscript and approved its final version.

## Supplementary Material

Additional file 1 **Principal component analysis on 19 climatic variables for spatially unique observations of *Ensatina *within California**. PC1 is responsible for 41.2% of the climatic variation and reflects wet and cold gradients (Mean Temperature of Coldest Quarter, Mean Temperature of Wettest Quarter, Min Temperature of Coldest Period, and Precipitation Seasonality; variables listed in decreasing order of importance). PC2 is responsible for 32.5% of the variation and reflects drier and warmer gradients (Mean Temperature of Warmest Quarter, Mean Temperature of Driest Quarter, Max Temperature of Warmest Period, Temperature Annual Range, and Temperature Seasonality; variables listed in decreasing order of importance). Colors and labels are in agreement with Figure 1; grey points refer to sampling within secondary contacts; lines demark 50% density ellipses. Click here for file

Additional file 2 **Collecting localities for allozymic data, sample sizes, and geographic location**. Underlined names represent localities at the center of the contact zones. Click here for file

Additional file 3 **Collecting localities for mtDNA data, geographic location and GenBank Accession numbers**. Click here for file
