# Supplementary figures and images for: Predictors for reproductive isolation in a ring species complex following genetic and ecological divergence
Source: BMC Evol Biol. 2011 Jul 6;11:194. doi: 10.1186/1471-2148-11-194 (PMC3225234; doi:10.1186/1471-2148-11-194)

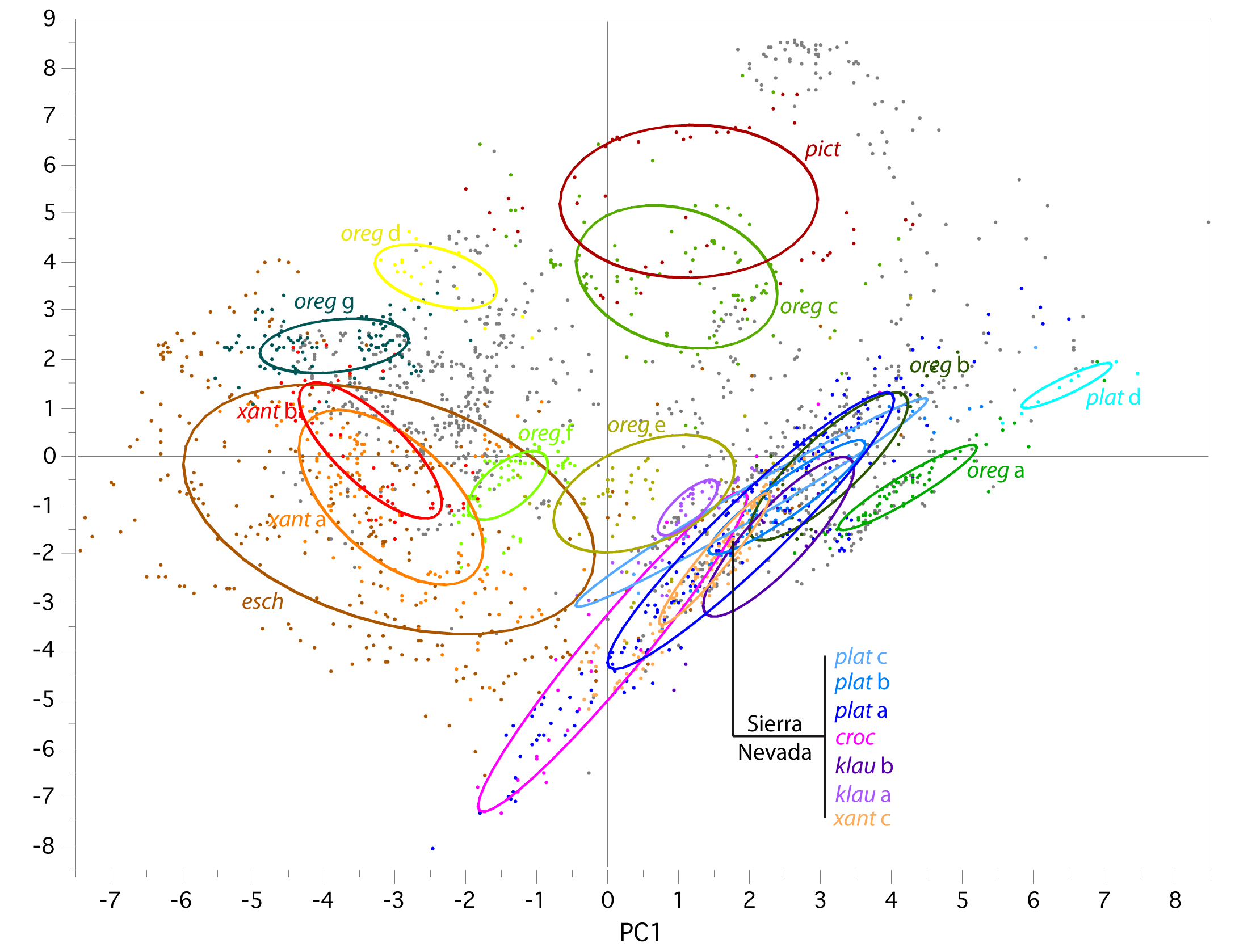

Supplement: Additional file 1 — Principal component analysis on 19 climatic variables for spatially unique observations of Ensatina within California. PC1 is responsible for 41.2% of the climatic variation and reflects wet and cold gradients (Mean Temperature of Coldest Quarter, Mean Temperature of Wettest Quarter, Min Temperature of Coldest Period, and Precipitation Seasonality; variables listed in decreasing order of importance). PC2 is responsible for 32.5% of the variation and reflects drier and warmer gradients (Mean Temperature of Warmest Quarter, Mean Temperature of Driest Quarter, Max Temperature of Warmest Period, Temperature Annual Range, and Temperature Seasonality; variables listed in decreasing order of importance). Colors and labels are in agreement with Figure 1; grey points refer to sampling within secondary contacts; lines demark 50% density ellipses. [file 1471-2148-11-194-S1.TIFF]
