# Supplementary material for: Predictors for reproductive isolation in a ring species complex following genetic and ecological divergence
Source: BMC Evol Biol. 2011 Jul 6;11:194. doi: 10.1186/1471-2148-11-194 (PMC3225234; doi:10.1186/1471-2148-11-194)
Supplement: Additional file 2 — Collecting localities for allozymic data, sample sizes, and geographic location. Underlined names represent localities at the center of the contact zones. [file 1471-2148-11-194-S2.PDF]

DataSet	Locality ID	subspecies	purity (80%)	cluster	*N*	Individual ID	Dec_Lat	Dec_Long
Sierra Nevada (Pereira and Wake, 2009)	Ishi Pishi Road	*oregonensis*	hybrid	*oreg c x oreg b*	11	MVZ 182066-76	41.371058	-123.493434
	Arcata	*picta*	parental	*pict*	10	MVZ 150430-7, MVZ 150439-40	40.877199	-124.066012
	Alderpoint	*oregonensis*	parental	*oreg c*	10	MVZ 158076-85	40.176726	-123.610266
	Salyer	*oregonensis*	hybrid	*pict x oreg b*	23	MVZ 215789-810, MVZ 215825	40.883229	-123.546940
	Little French Creek	*oregonensis*	parental	*oreg b*	8	MVZ 215811-8	40.770648	-123.306246
	Helena	*oregonensis*	hybrid	*oreg a x oreg b*	4	MVZ 217521, MVZ 215826-8	40.782537	-123.128305
	Oregon Mountain	*oregonensis*	hybrid	*oreg a x oreg b*	8	MVZ 215822-4, MVZ 233089-92, S#10990	40.745890	-122.973090
	Buckhorn Summit	*oregonensis*	hybrid	*oreg a x oreg b*	10	MVZ 215761-70	40.636559	-122.733265
	Hazel Creek	*oregonensis*	parental	*oreg a*	14	MVZ 215747-60	41.055008	-122.352126
	Ingot	*oregonensis*	parental	*oreg a*	8	MVZ 172523-5, MVZ 167974, MVZ 182000-3	40.726034	-122.078822
	Oak Run	*oregonensis*	parental	*oreg a*	6	MVZ 173211, MVZ 173213, MVZ 195575, MVZ 197518-9, MVZ 195571	40.653636	-121.942742
	Whitmore	*oregonensis*	parental	*oreg a*	4	MVZ 178769, MVZ 178772-3, MVZ 173215	40.646724	-121.815871
	Bear Creek	*oregonensis*	parental	*oreg a*	5	MVZ 215771-2, MVZ 215909, MVZ 218075, S#10995	40.520335	-121.910672
	Viola	*platensis*	parental	*plat d*	9	MVZ 211831-5, MVZ 217522-3, MVZ 217525, MVZ 215829	40.530006	-121.658350
	Bluff Springs	*platensis*	hybrid	*plat d x plat c*	7	MVZ 215788, MVZ 215830, MVZ 211838, MVZ 218076-7, S#10870-1	40.402150	-121.810901
	Potato Patch Campground	*platensis*	hybrid	*plat d x plat c*	6	MVZ 197523-5, MVZ 194143-4, MVZ 195574	40.192885	-121.531980
	Feather River	*platensis*	hybrid	*plat d x plat c*	11	MVZ 218062-72	39.684311	-121.313640

Bald Mountain	*platensis*	hybrid	*plat c x plat b*	7	MVZ 173177-83	39.335333	-121.014167
Yankee Jim	*platensis*	parental	*plat c*	10	MVZ 172463, MVZ 172466-8, MVZ 172474-9	39.037000	-120.907500
GeorgeTown	*platensis*	hybrid	*plat c x plat b*	4	MVZ 172441-4	38.926721	-120.764011
Blodgett	*platensis*	hybrid	*plat c x plat b*	12	MVZ 172450-8, MVZ 163821-2, S#5414	38.844150	-120.505990
West Panther Creek	*platensis*	hybrid	*plat c x plat b*	10	MVZ 195229-38	38.535201	-120.352750
Camp Connell	*platensis*	hybrid	*plat c x plat b*	10	MVZ 158054-63	38.323358	-120.263349
Holcomb Creek	*platensis*	parental	*plat b*	7	MVZ 195240-6	38.306015	-120.382151
Tuolumne	*platensis*	parental	*plat b*	5	MVZ 177864-8	37.999943	-120.175055
Tuolumne UB	*platensis*	parental	*plat b*	5	MVZ 158887-91	38.003040	-120.133485
Wagner Ridge	*platensis*	hybrid	*plat b x plat a*	6	MVZ 173167-72	37.785310	-120.155314
Westfall	*platensis*	parental	*plat a*	5	MVZ 173133-5, MVZ 173147, MVZ 173137	37.444344	-119.652110
Southfork	*platensis*	parental	*plat a*	2	MVZ 227233-4, MVZ	37.204731	-119.408554
Auberry	*platensis*	parental	*plat a*	4	MVZ 227220-1, MVZ 168882, S#486	37.122634	-119.369396
Hartland	*platensis*	parental	*plat a*	5	MVZ 168926-30	36.654723	-118.957292
Sugarloaf	*platensis*	parental	*plat a*	5	MVZ 157830-33	35.831630	-118.628263
Kern River	*platensis*	parental	*plat a*	10	MVZ 22722-8, MVZ 222475-8, S#10047-8	35.572657	-118.594460

North of San Francisco Bay	Barton Gulch	*oregonensis*	parental	*oreg d*	18	MVZ 168991-01, MVZ 169003-8, MVZ 169062		39.179368	-123.680855
(Pereira and Wake, 2009)	Sea Ranch	*oregonensis*	parental	*oreg d*	11	MVZ 181220-7		38.732701	-123.464184
			parental	*oreg d*		MVZ 167979-81		38.700260	-123.418549
	Orrs Spring	*oregonensis*	hybrid	*oreg d x oreg e*	8	S#4681-8		39.267812	-123.308184
	Branscomb Rd	*oregonensis*	hybrid	*oreg d x oreg c*	10	MVZ 189088-91		39.686337	-123.485194
			hybrid	*oreg d x oreg c*		MVZ 194080		39.659760	-123.684440
			hybrid	*oreg d x oreg c*		MVZ 194082-6		39.686337	-123.485194
	Drive-thru tree	*oregonensis*	parental	*oreg c*	22	MVZ 182016-37		39.858552	-123.718114
	Usal Rd	*oregonensis*	parental	*oreg c*	13	MVZ 189053-65		39.789726	-123.828270
	Longvale	*oregonensis*	hybrid	*oreg c x oreg e*	10	MVZ 182006-15		39.545482	-123.422717
	Maillard	*oregonensis*	hybrid	*oreg c x oreg e*	9	MVZ 195515-23		38.904776	-123.319921
	Parramore Crk	*oregonensis*	parental	*oreg e*	12	MVZ 194055		39.316723	-122.933390
			parental	*oreg e*		MVZ 194056		39.318102	-122.930207
			parental	*oreg e*		MVZ 194057-60		39.316551	-122.919643
			parental	*oreg e*		MVZ 194934-9		39.316792	-122.926406
	Cobb	*oregonensis*	parental	*oreg e*	16	MVZ 195524-39		38.831651	-122.731431
	Robert Louis Stevenson	*oregonensis*	parental	*oreg e*	20	MVZ 186602-4		38.660323	-122.596224
			parental	*oreg e*		MVZ 186605-8		38.667017	-122.595745
			parental	*oreg e*		MVZ 186609-10		38.660323	-122.596224

		parental	*oreg e*		MVZ 186611-12	38.667017	-122.595745
		parental	*oreg e*		MVZ 194160-3	38.660770	-122.596090
		parental	*oreg e*		MVZ 194138-41	38.663670	-122.598500
		parental	*oreg e*		MVZ 194142	38.665740	-122.597580
Samuel P Taylor	*oregonensis*	hybrid	*oreg e x oreg d*	8	MVZ 168522-3, MVZ 181295-300	38.023746	-122.726799
Geyser Rd	*oregonensis*	parental	*oreg e*	15	MVZ 194104	38.717129	-122.836922
		parental	*oreg e*		MVZ 194106	38.765352	-122.819546
		parental	*oreg e*		MVZ 194108	38.765454	-122.809002
		parental	*oreg e*		MVZ 194109	38.770117	-122.812076
		parental	*oreg e*		MVZ 194110-2	38.776369	-122.820422
		parental	*oreg e*		MVZ 194113	38.798955	-122.825207
		parental	*oreg e*		MVZ 194155-6	38.763695	-122.819283
		parental	*oreg e*		MVZ 194061	38.756686	-122.826136
		parental	*oreg e*		MVZ 194062	38.764696	-122.817701
		parental	*oreg e*		MVZ 194063-5	38.764072	-122.807948
Occidental	*oregonensis*	hybrid	*oreg e x oreg f*	18	MVZ 181309-26	38.412797	-122.950175
Calistoga Rd	*oregonensis*	parental	*oreg f*	20	MVZ 194149	38.518475	-122.601920
		parental	*oreg f*		MVZ 194150	38.519197	-122.597021
		parental	*oreg f*		MVZ 194151-2	38.520230	-122.593391
		parental	*oreg f*		MVZ 194153	38.519987	-122.589760
		parental	*oreg f*		MVZ 194154	38.519262	-122.588228

			parental	*oreg f*		MVZ 194089	38.521152	-122.625107
			parental	*oreg f*		MVZ 194090	38.521117	-122.623314
			parental	*oreg f*		MVZ 194091-3	38.518475	-122.601920
			parental	*oreg f*		MVZ 194094-5	38.518992	-122.600433
			parental	*oreg f*		MVZ 194096-100	38.519262	-122.588228
			parental	*oreg f*		MVZ 194101	38.516942	-122.584512
			parental	*oreg f*		MVZ 194102	38.519774	-122.573352
	Conn Creek	*oregonensis*	parental	*oreg f*	20	MVZ 181250-9, MVZ 181262-71	38.505378	-122.395308
	Mount Veeder	*oregonensis*	parental	*oreg f*	10	MVZ 158115-24	38.418960	-122.413180
	Phoenix Lake	*oregonensis*	parental	*oreg f*	10	MVZ 158091-98, MVZ 194146-7	37.950669	-122.571957
	Copeland Creek	*xanthoptica*	parental	*xant a*	12	MVZ 187207-8	38.338601	-122.596482
	Siesta Valley	*xanthoptica*	parental	*xant a*	12	MVZ 188984-91, S#4090-3	37.875897	-122.214785
San Francisco Bay	Copeland Creek	*xanthoptica*	parental	*xant a*	1	MVZ 189009	38.333765	-122.625014
(Pereira and Wake, 2009)	Sonoma Mountain Road	*xanthoptica*	parental	*xant a*	4	MVZ 205023-4	38.369662	-122.602998
			parental	*xant a*		MVZ 205011	38.369801	-122.597677
			parental	*xant a*		MVZ 205006	38.367179	-122.585632
	Parker Hill	*xanthoptica*	parental	*xant a*	19	MVZ 205681-99	38.485823	-122.696371
	Rieble Road	*xanthoptica*	parental	*xant a*	5	MVZ 208465	38.504240	-122.733323
			parental	*xant a*		MVZ 208467	38.504688	-122.732710

		parental	*xant a*		MVZ 208466	38.505997	-122.724568
		parental	*xant a*		MVZ 208448-9	38.509654	-122.716119
Trenton Healdsburg Road	*xanthoptica*	parental	*xant a*	11	MVZ 208459-61	38.487692	-122.853805
		parental	*xant a*		MVZ 223111-3, MVZ 223115-6, S#10776-7	38.494343	-122.863037
		parental	*xant a*		MVZ 215840	38.503828	-122.873254
Mirabel Road	*xanthoptica*	parental	*xant a*	6	MVZ 208400, MVZ 208407	38.473768	-122.905483
		parental	*xant a*		MVZ 208431	38.474251	-122.905045
		parental	*xant a*		MVZ 223053	38.473653	-122.901024
		parental	*xant a*		MVZ 223089-90	38.473619	-122.899272
Pressley Road	*oregonensis*	hybrid	*xant a x oreg f*	6	MVZ 205004	38.387290	-122.549407
		hybrid	*xant a x oreg f*		MVZ 205001	38.365453	-122.561971
		hybrid	*xant a x oreg f*		MVZ 204997	38.362348	-122.554458
		hybrid	*xant a x oreg f*		MVZ 205026-7	38.363777	-122.559366
		hybrid	*xant a x oreg f*		MVZ 204998	38.373043	-122.545392
South Russian River 1	*xanthoptica*	parental	*xant a*	15	MVZ 223094, MVZ 223098, MVZ 223102, MVZ 223105, S#10803	38.510383	-122.882041
North Russian River 1	*oregonensis*	parental	*oreg f*		MVZ 215782-4, MVZ 215775-9, MVZ 215885, MVZ 215887	38.510210	-122.882872
Pythian Road	*oregonensis*	parental	*oreg f*	15	MVZ 205668-76	38.449114	-122.572852
		parental	*oreg f*		MVZ 205642-3	38.446318	-122.573115
		parental	*oreg f*		MVZ 205479-82	38.448999	-122.572950
Mark West Springs Road	*oregonensis*	parental	*oreg f*	10	MVZ 208385	38.523724	-122.719165

		parental	*oreg f*		MVZ 205645-8, MVZ 205677-80	38.530301	-122.724169
		parental	*oreg f*		MVZ 208387	38.542279	-122.719302
Martinelli Road	*oregonensis*	parental	*oreg f*	8	MVZ 208434-9	38.505330	-122.918209
		parental	*oreg f*		MVZ 208384, MVZ 208394	38.496310	-122.915086
Green Hill Road	*oregonensis*	parental	*oreg f*	8	MVZ 208416	38.402766	-122.896815
		parental	*oreg f*		MVZ 208406, MVZ 208412	38.413952	-122.892628
		parental	*oreg f*		MVZ 208415	38.411263	-122.890967
		parental	*oreg f*		MVZ 208413	38.414857	-122.898532
		parental	*oreg f*		MVZ 208417	38.398630	-122.898038
		parental	*oreg f*		MVZ 208418	38.401560	-122.897864
		parental	*oreg f*		MVZ 208414	38.414030	-122.893332
North of Occidental	*oregonensis*	parental	*oreg f*	5	MVZ 181310, MVZ 181313, MVZ 181319, MVZ 181324, MVZ 181326	38.412797	-122.950175
North of Russian River 2	*oregonensis*	hybrid	*oreg f x oreg g*	6	MVZ 223058-9, MVZ 223061-4	38.514832	-122.888864
Los Alamos Road	*oregonensis*	hybrid	*oreg f x oreg g*	7	MVZ 204999	38.475316	-122.615145
		hybrid	*oreg f x oreg g*		MVZ 205517-8	38.474419	-122.616326
		hybrid	*oreg f x oreg g*		MVZ 205483	38.475316	-122.615145
		hybrid	*oreg f x oreg g*		MVZ 205485	38.474419	-122.616326
		hybrid	*oreg f x oreg g*		MVZ 205478	38.475316	-122.615145
		hybrid	*oreg f x oreg g*		MVZ 205486	38.470864	-122.618558

Coleman Valley Road	*oregonensis*	hybrid	*oreg f x oreg g*	7	MVZ 219740-6	38.407257	-122.986557
Wagnon Road	*oregonensis*	hybrid	*oreg f x oreg g*	4	MVZ 208425	38.377758	-122.891806
		hybrid	*oreg f x oreg g*		MVZ 208432	38.375739	-122.879199
		hybrid	*oreg f x oreg g*		MVZ 208422	38.382455	-122.888917
		hybrid	*oreg f x oreg g*		MVZ 208421	38.380072	-122.890187
Parrington Creek	*oregonensis*	parental	*oreg g*	4	MVZ 208396, MVZ 208401, MVZ 208403, MVZ 208409	38.413793	-122.929043
Bittner Road	*oregonensis*	parental	*oreg g*	7	MVZ 219720-4, MVZ 219726-7	38.395036	-122.960787
Taylor State Park	*oregonensis*	hybrid	*oreg g x xant b*	1	MVZ 181296	38.023746	-122.726799
Forest Knowlls	*oregonensis*	parental	*oreg g*	4	MVZ 219633-5, MVZ 219637	38.008143	-122.683235
Bolinas Road	*oregonensis*	parental	*oreg g*	6	MVZ 219627-32	37.972215	-122.603587
Phoenix Lake	*oregonensis*	hybrid	*oreg g x xant b*	2	MVZ 158096, MVZ 194147	37.950669	-122.571957
Pilarcitos Creek	*oregonensis*	parental	*oreg g*	2	MVZ 185818-9	37.494901	-122.421274
Tunitus Creek Road	*oregonensis*	hybrid	*oreg g x xant b*	8	MVZ 202342-9	37.414875	-122.349534
Skyline Blvd.	*oregonensis*	parental	*oreg g*	13	MVZ 167292-4, MVZ 167296, MVZ 158132, MVZ 158135-6, MVZ 158141-3	37.458008	-122.337219
		parental	*oreg g*		MVZ 158145-7	37.426757	-122.298127
Woodside	*xanthoptica*	hybrid	*oreg g x xant b*	8	MVZ 215831-6	37.398406	-122.260346
		hybrid	*oreg g x xant b*		MVZ 215837-8	37.312440	-122.313838
Canyon Rd.	ssp.	parental	*xant b*	3	MVZ 219652-3, MVZ 219655	37.212944	-122.333574
Pine Flat Rd.	ssp.	parental	*xant b*	3	MVZ 219661-3	37.079100	-122.140652

	Swanton Rd.	ssp.	parental	*xant b*	3	MVZ 219656-8	37.008705	-122.190197
	Cave Gulch	ssp.	parental	*xant b*	7	MVZ 186613-5, MVZ 186617-8, MVZ 187205, MVZ 186619	36.996440	-122.056757
	Santa Cruz Co. Line	*xanthoptica*	parental	*xant b*	10	MVZ 205520-2, MVZ 205524-5, MVZ 205527-31	36.997793	-121.685466
	Watsonville	*xanthoptica*	parental	*xant b*	10	MVZ 181378-82, MVZ 181436-40	36.979270	-121.710586
	Strawbwerry Rd.	*eschscholtzii*	parental	*esch*	6	MVZ 181386-7	36.832786	-121.686262
			parental	*esch*		MVZ 205701-4	36.830539	-121.677848
	Monterey City	*eschscholtzii*	parental	*esch*	3	MVZ 211877-9	36.600207	-121.892529
	Coast Rd.	*eschscholtzii*	parental	*esch*	10	MVZ 167456-7, MVZ 167460-1, MVZ 167463-8	36.331002	-121.861306
South of San Francisco Bay	Skyline Blvd.	*oregonensis*	parental	*oreg g*	19	MVZ 167292-300, MVZ 158131-40	37.458008	-122.337219
(Pereira and Wake, 2009)	Sanborn Canyon	*oregonensis*	parental	*oreg g*	16	MVZ 150445-60	37.250403	-122.094983
	Gazos Cr	*oregonensis*	parental	*oreg h*	7	S#4178-84	37.185761	-122.331534
	UC Santa Cruz	*xanthoptica*	parental	*xant b*	2	MVZ 158157-8	36.9964399	-122.0567572
	Santa Cruz	*xanthoptica*	parental	*xant b*	15	S#2418-32	36.993226	-122.033229
	Soquel	*xanthoptica*	parental	*xant b*	10	S#2287-94	37.005877	-121.952020
			parental	*xant b*		S#2295-6	36.993226	-122.033229
	Uvas Reservoir	*xanthoptica*	parental	*xant b*	2	S#4394-5	37.065925	-121.689941
	<u>Hazel Dell</u>	*xanthoptica*	hybrid	*xant a x xant b*	4	MVZ 167405-8	36.993515	-121.734362

	Laguna Cr	*xanthoptica*	hybrid	*xant a x xant b*	5	MVZ 167410-4	37.079376	-122.139621
	Pacheco Pass	*xanthoptica*	parental	*xant a*	2	MVZ 157586-7	37.070190	-121.211088
	Mt. Hamilton Rd	*xanthoptica*	parental	*xant a*	9	MVZ 158153-6	37.352936	-121.735412
			parental	*xant a*		MVZ 158149-52	37.331455	-121.684072
			parental	*xant a*		MVZ 158148	37.366479	-121.671384
	Moraga Canyon	*xanthoptica*	parental	*xant a*	13	S#4001-9, S#4219, S#4391-3	37.822097	-122.133666
	San Juan Cyn	*eschscholtzii*	parental	*esch*	13	MVZ 167432-4	36.796390	-121.470150
	Cienega Valley Rd	*eschscholtzii*	parental	*esch*	4	MVZ 157624-7	36.691334	-121.302690
	Carmel Valley Rd	*eschscholtzii*	parental	*esch*	10	MVZ 172342-5, S#3779-84	36.511982	-121.770913
	Hastings	*eschscholtzii*	parental	*esch*	4	MVZ 172282-5	36.412496	-121.592813
	Santa Monica Mts.	*eschscholtzii*	parental	*esch*	2	MVZ 168640-51	34.120000	-118.630000
	Honey spring Rd.	*eschscholtzii*	parental	*esch*	3	MVZ 168647-9	32.670000	-116.750000
	San Jacinto Mts	*eschscholtzii*	parental	*esch*	1	MVZ 168646	33.666839	-116.693016
South California	Hartland	*platensis*	parental	*plat a*	10	MVZ 168936-445	36.654723	-118.957292
(Pereira and Wake, 2009)	Kern River	*platensis*	parental	*plat a*	11	MVZ 227222-32	35.572657	-118.594460
	Breckenridge Mt.	*croceater*	hybrid	*plat a x croc*	9	MVZ 168920-5, MVZ 163681-2, MVZ 169032	35.461821	-118.586550

Piute Mts.	*croceater*	hybrid	*plat a x croc*	4	MVZ 158086	35.483190	-118.404840
	croceater	parental	*croc*		MVZ 158087-9	35.440488	-118.391370
Little Mutau Creek	*croceater*	parental	*croc*	4	MVZ 195604-7	34.652894	-119.025407
Clear Creek	*croceater*	parental	*croc*	3	MVZ 167624-6	35.241596	-118.618189
Cummings Valley	*croceater*	parental	*croc*	6	MVZ 168916-9, MVZ 169046, MVZ 168948	35.048330	-118.557140
Tejon Ranch	*croceater*	parental	*croc*	10	MVZ 168883-92	35.026099	-118.745260
Pine Mt.	*eschscholtzii*	parental	*esch*	3	MVZ 168875-7	34.643333	-119.323677
Millard Canyon	*eschscholtzii*	parental	*esch*	12	MVZ 181453-8, MVZ 181468-73	33.979796	-116.775832
Crystal Creek	*klauberi*	parental	*klau b*	10	MVZ 185820-2, MVZ 185824-30	34.371979	-116.928589
Santa Rosa Mt	*klauberi*	parental	*klau b*	10	MVZ 185831-40	33.542858	-116.490088
Juch Canyon	*klauberi*	parental	*klau a*	9	MVZ 181392-400	33.097545	-116.624539
Sawmill Canyon	Hybrid Zone 1	hybrid	*klau b x esch*	5	MVZ 185805-6, MVZ 185847-9	34.056828	-116.851536
San Jacinto	Hybrid Zone 2	hybrid	*klau b x esch*	13	MVZ 172528-31, MVZ 172533, MVZ 181960-4	33.803083	-116.730465
		hybrid	*klau b x esch*		MVZ 181938-40	33.803083	-116.730465
Palomar	Hybrid Zone 3	hybrid	*klau a x esch*	37	MVZ 172596-601, MVZ 181412	33.270000	-116.850000
		hybrid	*klau a x esch*		MVZ 181405, MVZ 181973-4, MVZ 185807	33.290000	-116.830000
		hybrid	*klau a x esch*		MVZ 181406-09, MVZ 181411	33.290000	-116.790000
		hybrid	*klau a x esch*		MVZ 178750-1	33.300000	-116.830000

			hybrid	*klau a x esch*		MVZ 181975	33.290000	-116.830000
			hybrid	*klau a x esch*		MVZ 181976-86, MVZ 181988-92	33.300000	-116.800000
			hybrid	*klau a x esch*		MVZ 185808-9	33.313676	-116.809583
	Cuyamaca Mts	Hybrid Zone 4	hybrid	*klau a x esch*	51	MVZ 178732-4, MVZ 181327-8, MVZ 181401-4, MVZ 181410, MVZ 181941-59, MVZ 181967-72	32.990000	-116.590000
			hybrid	*klau a x esch*		MVZ 178739	32.990400	-116.591110
			hybrid	*klau a x esch*		MVZ 181415-20	33.010000	-116.610000
			hybrid	*klau a x esch*		MVZ 167951-8, MVZ 169051	33.030000	-116.590000
southern Sierra Nevada	Yankee Jim	*platensis*	parental		10	S#5828-36, S#5838	39.037000	-120.907500
(new data)	Old Blodgett	*platensis*	parental		3	S#5890-1, S#5893	38.844150	-120.505990
	New Blodgett	*platensis*	parental		3	S#5408-9, S#5214	38.899936	-120.658350
	Georgetown	*platensis*	parental		8	S#5902-9	38.926721	-120.764011
	Wpanther Crk	*platensis*	parental		10	S#6918-27	38.535201	-120.352750
	Camp Connell	*platensis*	parental		10	S#6863-72	38.323358	-120.263349
	Holcomb Crk Rd	*platensis*	parental	*Nplat*	7	S#9450-6	38.306015	-120.382151
	ENE Tuolumne	*platensis*	parental	*Nplat*	5	S#7725-9	37.999943	-120.175055
	ENE Tuolumne UB	*platensis*	parental	*Nplat*	5	S#7326-30	38.003040	-120.133485
	Wagner Ridge	*platensis*	hybrid	*Nplat x plat a*	6	S#6308-13	37.785310	-120.155314
	Briceburg	*xanthoptica*	parental	*xant3*	2	S#10208-9	37.611333	-119.958843
	Cheapo	*xanthoptica*	parental	*xant3*	5	S#6237-40, S#6249	37.348779	-119.592265

	Saddle							
	Westfall	*platensis*	parental	*plat a*	5	S#6215-6, S#6217A, S#6217B, S#6219	37.444344	-119.652110
	Jose Basin	*platensis*	parental	*plat a*	2	S#3985-6	37.133370	-119.370490
	Kings Rd	*platensis*	parental	*plat a*	1	S#9891	36.878971	-119.121938
	Auberry	*platensis*	parental	*plat a*	2	S#8852-3	37.122634	-119.369396
	ESE Southfork	*platensis*	parental	*plat a*	2	S#8855-6	37.204731	-119.408554
	Old Hartland	*platensis*	parental	*plat a*	5	S#4029-33	36.654723	-118.957292
	Sugarloaf	ssp.	hybrid	*plat a x croc*	5	K3135, K3143, K3145-7	35.831630	-118.628263
	Cowflat a	*croceater*	parental	*croc*	7	S#9726-32	35.505918	-118.691320
	Cowflat b	*croceater*	parental	*croc*	1	S#10446	35.446803	-118.787053
	Old Piute	*croceater*	parental	*croc*	4	S#6593-6	35.483190	-118.404840
	New Piute	*croceater*	parental	*croc*	3	S#10019-21	35.455869	-118.358306
	Kern River	*croceater*	parental	*croc*	10	S#9920, S#10024-8, S#10447-8, S#8690-1	35.572657	-118.594460
mid-ring contact	<u>Avery 1981</u>	Hybrid Zone	hybrid	*xant3 x plat b*	21	S#8678-85, S#8788-9, S#8908, S#9099, S#9133, S#9140-2, S#9148, S#9325-6, S#9366-7	38.211060	-120.373230
(Alexandrino et al., 2009)					7	S#8686, S#8794-6, S#8909, S#9574, S#9679	38.212285	-120.368416
					7	S#8689-93, S#8910, S#9098		
					12	S#9132, S#9135-9, S#9145, S#9147, S#9149-50, S#9327, S#9681	38.201320	-120.368360
					16	S#7720, S#7768-9, S#7941, S#7949, S#8042, S#8688, S#8798, S#8905, S#9144, S#9146, S#9152a-b, S#9324, S#9328, S#9365	38.213286	-120.368896

				2	S#6748-9	38.213348	-120.377131
				6	S#5787-91, S#8043	38.197270	-120.361470
				25	S#5796, S#5855, S#6394-7, S#6738-40, S#6774, S#6780, S#6858, S#7170-1, S#7314-7, S#7743, S#7950, S#7970-2, S#8044-5	38.191430	-120.356050
				11	S#6747, S#6765, S#7172, S#7321, S#7498, S#7723, S#7787-8, S#7966, S#7977-8	38.203000	-120.357220
				17	S#7181-4, S#7324-5, S#7333, S#7366, S#7722, S#7745, S#7773-4, S#7942, S#7948-9, S#8047-8,	38.209692	-120.388686
				24	S#6733-7, S#6766-7, S#6859, S#7322-3, S#7367, S#7744, S#7775-9, S#7943-4, S#7946, S#7951, S#7965, S#7980-1	38.207829	-120.390257
				10	S#6778-9, S#7368, S#7741, S#7998-8000, S#9680, S#9682, S#9914	38.205966	-120.390215
				33	S#6404-5, S#6741-6, S#6763-4, S#6776, S#6897, S#7173-80, S#7369-70, S#7497, S#7742a-b, S#7781-4, S#7940, S#7973-4, S#8046	38.199930	-120.357570
				9	S#6777, S#7318-20, S#7785-6, S#7975-6, S#8455	38.199250	-120.358570
West Point 1981	Hybrid Zone	hybrid	*xant3 x plat b*	6	S#7304-5, S#7340-3	38.410735	-120.591895
				5	S#6900, S#6930-3	38.405284	-120.593025
				4	S#6901, S#6934-6	38.400108	-120.588914
				3	S#6698, S#7345-6	38.399315	-120.588652
				12	S#7306-10, S#7344, S#7347-52	38.398694	-120.588476
				4	S#7353, S#6696-7, S#6902	38.396486	-120.588649
				3	S#6843, S#6937-8	38.394795	-120.588822
				6	S#6695, S#6903, S#6939-40, S#7311, S#7354	38.393726	-120.589432
				7	S#6841-2, S#6904-6, S#6941, S#7355	38.392864	-120.589562
				2	S#6907, S#7356	38.391760	-120.590347

				8	S#6694, S#6838-40, S#6942-5	38.390172	-120.588249
				2	S#6946-7	38.388688	-120.586893
				1	S#6908	38.386267	-120.569334
West Point 2000	Hybrid Zone	hybrid	*xant3 x plat b*	1	JA98	38.410330	-120.591930
				8	JA53-5, JA146-9, JA183	38.399936	-120.589176
				3	JA18, JA91-2	38.399510	-120.588430
				1	JA16	38.398650	-120.588440
				3	JA22, JA93, JA150	38.396040	-120.589010
				8	JA5, JA23, JA56, JA95, JA155-7, JA184	38.394430	-120.589140
				10	JA27, JA57, JA94, JA151-4, JA185-7	38.393340	-120.589310
				9	JA24, JA158-63, JA188-9	38.392440	-120.589720
				14	JA6, JA15, JA25, JA58-9, JA96-7, JA164-5, JA190-4	38.391540	-120.590110
				3	JA167-8, JA196	38.390172	-120.588249
