# Supplementary material for: Predictors for reproductive isolation in a ring species complex following genetic and ecological divergence
Source: BMC Evol Biol. 2011 Jul 6;11:194. doi: 10.1186/1471-2148-11-194 (PMC3225234; doi:10.1186/1471-2148-11-194)
Supplement: Additional file 3 — Collecting localities for mtDNA data, geographic location and GenBank Accession numbers. [file 1471-2148-11-194-S3.PDF]

sample ID	population	lat	long	GenBank
202330	croc	35.04722	-118.48598	L75796
195607	croc	34.65289	-119.02541	L75797
172480	plat c	39.037	-120.9075	JN022615
225030	plat c	39.01371	-120.33931	JN022616
172459	oreg b	38.9064	-120.6445	L75813
CM165	oreg b	40.90261	-123.58649	JN022617
CM166	oreg b	40.90261	-123.58649	JN022618
CM167	oreg b	40.90261	-123.58649	JN022619
CM168	oreg b	40.90261	-123.58649	JN022620
CM171	oreg b	40.90261	-123.58649	JN022621
CM193	oreg b	40.90261	-123.5826	JN022622
RP212	oreg b	40.80391	-123.47127	JN022623
RP213	oreg b	40.80391	-123.47127	JN022624
RP214	oreg b	40.80391	-123.47127	JN022625
RP216	oreg b	40.94155	-123.63504	JN022626
RP217	oreg b	40.94155	-123.63504	JN022627
RP223	oreg b	40.76243	-123.88557	JN022628
237526	oreg b	40.9394	-123.624	FJ151673
185823	klau b	34.37222	-116.92694	L75801
185844	klau b	33.54286	-116.49009	L75803
173221	plat d	40.26338	-121.40098	JN022629
194145	plat d	40.2024967	-121.512887	JN022630
197517	plat d	40.188367	-121.5319426	JN022631
214427	plat d	40.3312474	-121.7307627	JN022632
234865	plat d	40.51857	-121.70877	JN022633
215993	plat b	38.2690621	-120.3272568	JN022634
219619	plat b	38.3233577	-120.2633487	JN022635
223033	plat b	38.336902	-120.179976	JN022636
225733	plat b	38.336902	-120.179976	JN022637
158006	plat b	38.275	-120.337	L75812
194896	oreg d	39.1526	-123.54166	JN022638
237548	oreg d	39.39033	-123.23542	JN022639

194081	oreg d	39.68634	-123.48519	L75806
237548	oreg d	39.39033	-123.23542	FJ151799
237547	oreg d	39.3542	-123.65803	FJ151800
194896	oreg d	39.1526	-123.54166	L75809
223052	oreg e	38.7968059	-122.7022426	JN022640
211897	oreg g	38.4167	-122.9167	FJ151720
208426	oreg g	38.4	-122.8667	FJ151721
237565	oreg g	38.367	-122.85	FJ151722
237558	oreg g	38.345	-122.861	FJ151736
SK71a	oreg g	38.1792	-122.9474	FJ151748
SK71b	oreg g	38.1792	-122.9474	FJ151749
237144	oreg g	38.1047	-122.8384	FJ151751
237145	oreg g	38.1047	-122.8384	FJ151752
SK74d	oreg g	38.0992	-122.8793	FJ151756
SK74e	oreg g	38.095	-122.884	FJ151757
SK75a	oreg g	38.0935	-122.8825	FJ151758
SK75b	oreg g	38.0935	-122.8825	FJ151759
SK75d	oreg g	38.0935	-122.8825	FJ151761
SK75e	oreg g	38.0935	-122.8825	FJ151762
SK75f	oreg g	38.0883	-122.8807	FJ151763
SK75g	oreg g	38.0883	-122.8807	FJ151764
SK75h	oreg g	38.0883	-122.8807	FJ151765
SK75i	oreg g	38.0883	-122.8807	FJ151766
SK75j	oreg g	38.0883	-122.8807	FJ151767
SK76a	oreg g	38.0234	-122.8689	FJ151768
SK76b	oreg g	38.0234	-122.8689	FJ151769
219633	oreg g	38.00814	-122.68323	FJ151770
219634	oreg g	38.00814	-122.68323	FJ151771
SK79a	oreg g	37.9697	-122.6982	FJ151773
SK79b	oreg g	37.9697	-122.6982	FJ151774
237491	oreg g	37.88854	-122.44851	FJ151775
237492	oreg g	37.88854	-122.44851	FJ151776
185818	oreg g	37.4949	-122.42127	FJ151777

222995	oreg g	37.45801	-122.33722	FJ151778
230800	oreg g	37.42927	-122.28945	FJ151779
230782	oreg g	37.34417	-122.33917	FJ151781
230783	oreg g	37.34417	-122.33917	FJ151782
222987	oreg g	37.3151	-122.186	FJ151783
222988	oreg g	37.3151	-122.186	FJ151784
222989	oreg g	37.3151	-122.186	FJ151785
222990	oreg g	37.3151	-122.186	FJ151786
222991	oreg g	37.3151	-122.186	FJ151787
222992	oreg g	37.3151	-122.186	FJ151788
230785	oreg g	37.30528	-122.26694	FJ151789
230786	oreg g	37.28556	-122.28417	FJ151790
230889	oreg g	37.29167	-122.28056	FJ151791
230890	oreg g	37.29167	-122.28056	FJ151792
230891	oreg g	37.29167	-122.28056	FJ151793
230892	oreg g	37.29167	-122.28056	FJ151794
230893	oreg g	37.29167	-122.28056	FJ151795
230894	oreg g	37.29167	-122.28056	FJ151796
230895	oreg g	37.29167	-122.28056	FJ151797
230838	oreg g	37.21131	-122.15092	FJ151798
194159	oreg f	38.7	-122.8333	FJ151693
158159	oreg f	38.6833	-123.1083	FJ151694
238155	oreg f	38.64148	-122.59617	FJ151695
208381	oreg f	38.625	-122.775	FJ151696
221138	oreg f	38.6167	-123.1583	FJ151697
217497	oreg f	38.5833	-122.925	FJ151698
237529	oreg f	38.60488	-122.84617	FJ151700
237530	oreg f	38.60488	-122.84617	FJ151701
238160	oreg f	38.60272	-122.64426	FJ151702
238157	oreg f	38.58413	-122.6198	FJ151703
237531	oreg f	38.58333	-122.69417	FJ151704
226755	oreg f	38.575	-122.767	FJ151705
226752	oreg f	38.55	-122.617	FJ151706

226051	oreg f	38.54222	-122.71917	FJ151707
188992	oreg f	38.50527	-122.39684	FJ151708
221126	oreg f	38.517	-122.933	FJ151710
221086	oreg f	38.51667	-122.91667	FJ151711
226053	oreg f	38.50806	-122.88333	FJ151712
226054	oreg f	38.50944	-122.88389	FJ151713
188994	oreg f	38.50527	-122.39684	FJ151714
226745	oreg f	38.473	-122.909	FJ151715
158111	oreg f	38.43349	-122.24253	FJ151716
205516	oreg f	38.4667	-122.6167	FJ151717
226760	oreg f	38.44778	-122.92472	FJ151718
158112	oreg f	38.43349	-122.24253	FJ151719
205514	oreg f	38.375	-122.458	FJ151723
237500	oreg f	38.36871	-122.41083	FJ151724
237501	oreg f	38.35919	-122.38616	FJ151725
237495	oreg f	38.35533	-122.38274	FJ151726
237496	oreg f	38.35757	-122.38451	FJ151727
237497	oreg f	38.35757	-122.38451	FJ151728
237498	oreg f	38.35757	-122.38451	FJ151729
237499	oreg f	38.35757	-122.38451	FJ151730
237502	oreg f	38.35643	-122.3837	FJ151731
238159	oreg f	38.35757	-122.38451	FJ151732
237516	oreg f	38.34754	-122.41921	FJ151733
237519	oreg f	38.34512	-122.39843	FJ151734
237523	oreg f	38.34512	-122.39843	FJ151735
237515	oreg f	38.34416	-122.41312	FJ151738
237517	oreg f	38.34464	-122.4099	FJ151739
237518	oreg f	38.34416	-122.40318	FJ151740
237513	oreg f	38.34174	-122.39271	FJ151741
237520	oreg f	38.33457	-122.38029	FJ151742
237521	oreg f	38.3345	-122.37523	FJ151743
237522	oreg f	38.3345	-122.37523	FJ151744
237494	oreg f	38.3209	-122.3455	FJ151745

237549	oreg f	38.29623	-122.39847	FJ151746
237532	oreg f	38.29478	-122.39812	FJ151747
150876	oreg c	?	?	JN022641
181357	oreg c	40.1044023	-123.9787194	JN022642
220589	oreg c	40.5285766	-124.0347339	JN022643
DBW6576	oreg c	40.51059	-124.1608	JN022644
DBW6578	oreg c	41.73509	-123.98396	JN022645
DBW6579	oreg c	39.85828	-123.71973	JN022646
DBW6586	oreg c	39.55567	-123.41814	JN022647
220589	oreg c	40.705	-122.909	FJ151688
RP215	oreg a	40.69984	-122.91679	JN022648
197522	oreg a	40.88276	-121.72091	FJ151680
SRK 19693	oreg a	40.8738	-122.2543	FJ151681
SRK 19713	oreg a	40.8785	-122.2845	FJ151682
182000	oreg a	40.77578	-122.00388	L75808
SRK 1970	oreg a	40.7291	-122.3079	FJ151684
CM148	pict	40.66771	-123.92365	JN022649
CM149	pict	40.66771	-123.92365	JN022650
CM150	pict	40.66771	-123.92365	JN022651
CM186	pict	40.76243	-123.88557	JN022652
CM187	pict	40.76243	-123.88557	JN022653
CM188	pict	40.76134	-124.0584	JN022654
DBW6541	pict	40.8711	-124.07242	JN022655
DBW6542	pict	40.8711	-124.07242	JN022656
DBW6543	pict	40.8711	-124.07242	JN022657
DBW6544	pict	40.8711	-124.07242	JN022658
DBW6546	pict	40.8711	-124.07242	JN022659
DBW6548	pict	40.8711	-124.07242	JN022660
DBW6549	pict	40.8711	-124.07242	JN022661
DBW6552	pict	40.9295	-123.85925	JN022662
DBW6554	pict	40.9295	-123.85925	JN022663
DBW6555	pict	40.9295	-123.85925	JN022664
DBW6556	pict	40.9295	-123.85925	JN022665

DBW6557	pict	40.9295	-123.85925	JN022666
DBW6558	pict	40.9295	-123.85925	JN022667
DBW6560	pict	40.9295	-123.85925	JN022668
DBW6561	pict	40.9295	-123.85925	JN022669
DBW6563	pict	40.9295	-123.85925	JN022670
DBW6566	pict	40.89894	-123.92485	JN022671
DBW6567	pict	40.89894	-123.92485	JN022672
RP210	pict	40.98335	-124.0593	JN022673
RP209	pict	40.98335	-124.0593	JN022674
RP211	pict	40.98335	-124.0593	JN022675
RP221	pict	40.94155	-123.63504	JN022676
RP226	pict	40.76134	-124.0584	JN022677
RP236	pict	40.65468	-123.86491	JN022678
RP239	pict	40.65468	-123.86491	JN022679
RP240	pict	40.65468	-123.86491	JN022680
RP241	pict	40.76134	-124.0584	JN022681
RP242	pict	40.76134	-124.0584	JN022682
220597	pict	40.9099	-123.8673	FJ151674
195651	pict	40.75823	-123.87429	FJ151678
232660	esch	36.917	-121.667	FJ151951
195674	esch	36.85724	-121.59588	FJ151952
205706	esch	36.80216	-121.49906	FJ151953
232663	esch	36.5855	-121.86767	FJ151955
233081	esch	36.5855	-121.86767	FJ151956
232661	esch	36.575	-121.875	FJ151957
167462	esch	36.331	-121.86131	FJ151958
237493	esch	35.885	-121.41194	FJ151962
167654	esch	34.79473	-120.04545	L75799
181460	esch	33.9798	-116.77583	L75798
237147	esch	32.94351	-116.63731	FJ151963
178729	esch	32.83	-116.68	L75800
191684	Sklau	32.99	-116.59	L75804
194908	Sklau	32.99	-116.59	L75805

173112	plat a	38.3428979	-120.3961373	JN022683
RP128	plat a	38.10536	-120.12918	JN022684
RP130	plat a	38.15007	-120.05927	JN022685
RP315	plat a	?	?	JN022686
237148	plat a	37.9341	-119.96097	FJ151969
237149	plat a	37.9341	-119.96097	FJ151970
237150	plat a	37.79806	-119.09975	FJ151972
157796	plat a	37.79528	-120.18222	FJ151973
157798	plat a	37.77333	-120.18389	FJ151975
157490	plat a	37.76528	-120.135	FJ151976
157491	plat a	37.75472	-120.11722	FJ151977
237152	plat a	37.74303	-119.76953	FJ151978
237160	plat a	37.73275	-119.61047	FJ151979
237157	plat a	37.72722	-119.54278	FJ151980
237161	plat a	37.72156	-119.62575	FJ151982
237162	plat a	37.72156	-119.62575	FJ151983
237155	plat a	37.71511	-119.65111	FJ151984
237156	plat a	37.71511	-119.65111	FJ151985
237154	plat a	37.69303	-119.76758	FJ151987
223080	plat a	37.432754	-119.584968	JN022687
237131	plat a	36.719	-118.92975	JN022688
237143	plat a	36.46538	-118.64769	JN022689
243285	plat a	37.51627	-119.84603	JN022690
243299	plat a	37.5022	-119.60635	JN022691
243310	plat a	37.53466	-119.65811	JN022692
243315	plat a	37.53976	-119.64956	JN022693
244092	plat a	37.3819	-119.6096	JN022694
244093	plat a	37.3805	-119.6117	JN022695
237289	plat a	37.61364	-119.68347	FJ151988
237292	plat a	37.54031	-119.65375	FJ151989
169033	plat a	37.49503	-119.63164	L75816
244095	plat a	37.3961	-119.6253	FJ151991
244096	plat a	37.3961	-119.6253	FJ151992

244097	plat a	37.3966	-119.6277	FJ151993
244098	plat a	37.3966	-119.6277	FJ151994
244092	plat a	37.3819	-119.6096	FJ151995
244093	plat a	37.3805	-119.6119	FJ151996
244094	plat a	37.3805	-119.6119	FJ151997
237128	plat a	36.77047	-118.70742	FJ151998
237287	plat a	36.71394	-118.98439	FJ151999
237130	plat a	36.69583	-118.91317	FJ152000
169165	plat a	36.65472	-118.95729	L75814
237168	plat a	36.54611	-118.89694	FJ152001
237175	plat a	36.38222	-118.87	FJ152002
208454	xant a	38.55	-122.7667	FJ151839
208456	xant a	38.508	-122.742	FJ151840
237577	xant a	38.5072	-122.88	FJ151841
226750	xant a	38.485	-122.844	FJ151842
205681	xant a	38.483	-122.7	L75819
205686	xant a	38.483	-122.7	FJ151843
226761	xant a	38.467	-122.858	FJ151844
217519	xant a	38.45	-122.7083	FJ151845
208457	xant a	38.4417	-122.85	FJ151846
237579	xant a	38.422	-122.832	FJ151847
237576	xant a	38.41389	-122.82833	FJ151848
215907	xant a	38.3833	-122.6417	FJ151849
237587	xant a	38.3661	-122.5675	FJ151851
237586	xant a	38.36528	-122.57639	FJ151852
237584	xant a	38.35528	-122.5425	FJ151853
237581	xant a	38.3323	-122.5828	FJ151854
222976	xant a	38.33043	-122.35373	FJ151855
237511	xant a	38.31476	-122.36382	FJ151856
237512	xant a	38.31476	-122.36382	FJ151857
181413	xant a	38.31152	-122.34883	FJ151858
237503	xant a	38.31186	-122.34942	FJ151859
237505	xant a	38.3131	-122.35029	FJ151860

237506	xant a	38.31352	-122.3516	FJ151861
237507	xant a	38.31352	-122.35492	FJ151862
237508	xant a	38.31262	-122.36007	FJ151863
237509	xant a	38.31228	-122.36077	FJ151864
237510	xant a	38.31228	-122.36077	FJ151865
237585	xant a	38.30962	-122.60939	FJ151866
237588	xant a	38.3084	-122.5488	FJ151867
237589	xant a	38.3084	-122.5488	FJ151868
237582	xant a	38.30217	-122.62268	FJ151869
237583	xant a	38.30217	-122.62268	FJ151870
237574	xant a	37.8984	-122.2216	FJ151872
237566	xant a	37.8918	-122.2175	FJ151873
237567	xant a	37.8918	-122.2175	FJ151874
237568	xant a	37.8918	-122.2175	FJ151875
237569	xant a	37.8918	-122.2175	FJ151876
237571	xant a	37.8918	-122.2175	FJ151877
237572	xant a	37.8918	-122.2175	FJ151878
237573	xant a	37.8918	-122.2175	FJ151879
163850	xant a	37.87184	-122.17544	L75818
215743	xant a	37.7181	-121.69975	FJ151880
CAS 207482	xant a	37.692	-122.45	FJ151881
215891	xant a	37.6833	-121.95	FJ151882
167291	xant a	37.55555	-122.33541	FJ151883
230770	xant a	36.9705	-121.41025	FJ151884
230866	xant b	37.192	-122.192	FJ151898
230867	xant b	37.192	-122.192	FJ151899
230816	xant b	37.183	-121.967	FJ151900
230787	xant b	37.183	-122.117	FJ151901
230870	xant b	37.175	-122.192	FJ151902
230871	xant b	37.175	-122.192	FJ151903
230872	xant b	37.175	-122.192	FJ151904
230873	xant b	37.175	-122.192	FJ151905
230877	xant b	37.175	-122.192	FJ151906

230878	xant b	37.175	-122.192	FJ151907
230879	xant b	37.175	-122.192	FJ151908
230880	xant b	37.175	-122.192	FJ151909
230881	xant b	37.175	-122.192	FJ151910
230882	xant b	37.175	-122.192	FJ151911
230883	xant b	37.175	-122.192	FJ151912
230884	xant b	37.175	-122.192	FJ151913
230868	xant b	37.158	-122.2	FJ151915
230869	xant b	37.158	-122.2	FJ151916
230818	xant b	37.125	-121.942	FJ151918
230819	xant b	37.125	-121.942	FJ151919
230821	xant b	37.125	-121.942	FJ151920
230822	xant b	37.125	-121.942	FJ151921
230823	xant b	37.125	-121.942	FJ151922
230824	xant b	37.125	-121.942	FJ151923
230825	xant b	37.125	-121.942	FJ151924
230826	xant b	37.125	-121.942	FJ151925
230827	xant b	37.125	-121.942	FJ151926
230828	xant b	37.125	-121.942	FJ151927
230829	xant b	37.125	-121.942	FJ151928
230874	xant b	37.117	-121.942	FJ151929
230805	xant b	37.04611	-121.75194	FJ151930
230814	xant b	37.01444	-121.71667	FJ151933
230832	xant b	37.01444	-121.71667	FJ151935
230833	xant b	37.01444	-121.71667	FJ151936
230834	xant b	37.01444	-121.71667	FJ151937
230835	xant b	37.01444	-121.71667	FJ151938
230836	xant b	37.01444	-121.71667	FJ151939
226739	xant b	37.00379	-121.67847	FJ151941
226740	xant b	37.00379	-121.67847	FJ151942
226742	xant b	37.00379	-121.67847	FJ151944
226743	xant b	37.00379	-121.67847	FJ151945
230839	xant b	36.99472	-121.74361	FJ151947

230887	xant b	36.9913	-121.69943	FJ151948
230888	xant b	36.9913	-121.69943	FJ151949
243224	xant c	38.3629	-120.54672	FJ151885
243142	xant c	38.35962	-120.59322	FJ151886
243143	xant c	38.35962	-120.59322	FJ151887
243145	xant c	38.35962	-120.59322	FJ151888
243217	xant c	38.35047	-120.54497	FJ151889
202316	xant c	38.1729	-120.464	L75817
236231	xant c	37.76889	-120.18917	FJ151891
236233	xant c	37.73778	-120.14917	FJ151892
244099	xant c	37.395	-119.6302	FJ151893
244100	xant c	37.395	-119.6302	FJ151894
244101	xant c	37.395	-119.6302	FJ151895
244091	xant c	37.305	-119.6474	FJ151897
